# Is Nitric Oxide Decrease Observed with Naphthoquinones in LPS Stimulated RAW 264.7 Macrophages a Beneficial Property?

**DOI:** 10.1371/journal.pone.0024098

**Published:** 2011-08-26

**Authors:** Brígida R. Pinho, Carla Sousa, Patrícia Valentão, Paula B. Andrade

**Affiliations:** REQUIMTE/Laboratório de Farmacognosia, Departamento de Química, Faculdade de Farmácia, Universidade do Porto, Porto, Portugal; Center for Disease Control and Prevention, United States of America

## Abstract

The search of new anti-inflammatory drugs has been a current preoccupation, due to the need of effective drugs, with less adverse reactions than those used nowadays. Several naphthoquinones (plumbagin, naphthazarin, juglone, menadione, diosquinone and 1,4-naphthoquinone), plus *p*-hydroquinone and *p*-benzoquinone were evaluated for their ability to cause a reduction of nitric oxide (NO) production, when RAW 264.7 macrophages were stimulated with lipopolysaccharide (LPS). Dexamethasone was used as positive control. Among the tested compounds, diosquinone was the only one that caused a NO reduction with statistical importance and without cytotoxicity: an IC_25_ of 1.09±0.24 µM was found, with 38.25±6.50% (*p*<0.001) NO reduction at 1.5 µM. In order to elucidate if this NO decrease resulted from the interference of diosquinone with cellular defence mechanisms against LPS or to its conversion into peroxynitrite, by reaction with superoxide radical formed by naphthoquinones redox cycling, 3-nitrotyrosine and superoxide determination was also performed. None of these parameters showed significant changes relative to control. Furthermore, diosquinone caused a decrease in the pro-inflammatory cytokines: tumour necrosis factor-alpha (TNF-α) and interleukin 6 (IL-6). Therefore, according to the results obtained, diosquinone, studied for its anti-inflammatory potential for the first time herein, has beneficial effects in inflammation control. This study enlightens the mechanisms of action of naphthoquinones in inflammatory models, by checking for the first time the contribution of oxidative stress generated by naphthoquinones to NO reduction.

## Introduction

Naphthoquinones are secondary metabolites widely distributed in nature. They are present mainly in several families of higher plants, as *Diospyros* spp., including the species producing the edible fruit persimmon, but also in fungi, lichens and in bacteria [1]. Naphthoquinones present a diversity of structures, exhibiting several substituents and can group together forming dimers, trimers and more seldom tetramers [2]. This chemical diversity probably explains the several activities described for them. Naphthoquinones exhibit very interesting pharmacological properties, being *Diospyros* extracts widely used in African, Chinese and Indian traditional medicine [1]. In particular, *Diospyros chamaethamnus* is characterized by the presence of several 7-methyljuglone derivatives, including five dimers (diospyrin, isodiospyrin, diosquinone, mamegakinone and biramentaceone), a trimer (xylospyrin) and a tretramer (6-[2-(7-methyljuglonyl)]-isoxylospyrin) [3].

Antibacterial [4], antimalarial [5], antipyretic [6] and antitumor [7] activities are some of the properties attributed to naphthoquinones. Although naphthoquinones have also a toxicological potential, mainly due to their ability to induce oxidative stress [8], they can be good lead compounds for new anti-inflammatory drugs, as several previous studies evidence. Bisnaphthoquinones inhibit platelet aggregation [9], plumbagin inhibits cytokines release [10], shikonin derivatives inhibit cyclooxygenase-2 expression [11], mast cell degranulation [12] and iNOS (an inducible calcium-independent isoform of nitric oxide (NO) synthases) [13], interfering with NO production [14]. However more studies are needed to confirm a really beneficial property.

During inflammation highly reactive species, such as superoxide radical, peroxynitrite, hydrogen peroxide, hypochlorous acid and NO are produced. NO is a small diffusible molecule with important biological functions, including vasodilatation, neurotransmission and inflammation. NO generated by iNOS in activated macrophages is important for host defences. NO modulates the synthesis of prostaglandins, tromboxans and other inflammatory molecules [15].

Macrophages are important producers of pro-inflammatory cytokines, such as tumour necrosis factor-alpha (TNF-α), interleukin 1β (IL-1β) and interleukin 6 (IL-6), when they are stimulated by an aggression, like lipopolysaccharide (LPS) exposure [16]. These pro-inflammatory cytokines allow an increase in blood flow and permeability into capillaries, leading to infiltration of immune cells. TNF-α has a central role in the inflammatory and destructive processes found in several human autoimmune and chronic inflammatory diseases [17]. IL-1β is important for the initiation and enhancement of inflammatory response to proliferation of some microorganisms [18] and IL-6 is regarded as an endogenous mediator of LPS-induced fever [19]. Furthermore, there is some evidence of the involvement of these cytokines in carcinogenicity [20].

Although inflammatory mediators like cytokines are necessary for successful defence against foreign invaders, their production also results in collateral damage to host tissue components, including proteins, lipids/membranes and DNA. A delicate balance between formation and detoxification of reactive species produced in inflammation process allows directing signalling pathways to physiological or pathological conditions. Therefore, inflammation plays a pivotal role in the pathogenesis and development of some diseases, like certain cancers, neurodegenerative lesions and chemical toxicity [21].

For the management of inflammation a broad range of immunosuppressive drugs, as calcineurin inhibitors, steroids and anti-inflammatory non-steroids have been used. Nevertheless, these drugs have undesirable side effects like metabolic derangements, development of infections, cancers and gastric toxicity [10].

**Figure 1:**
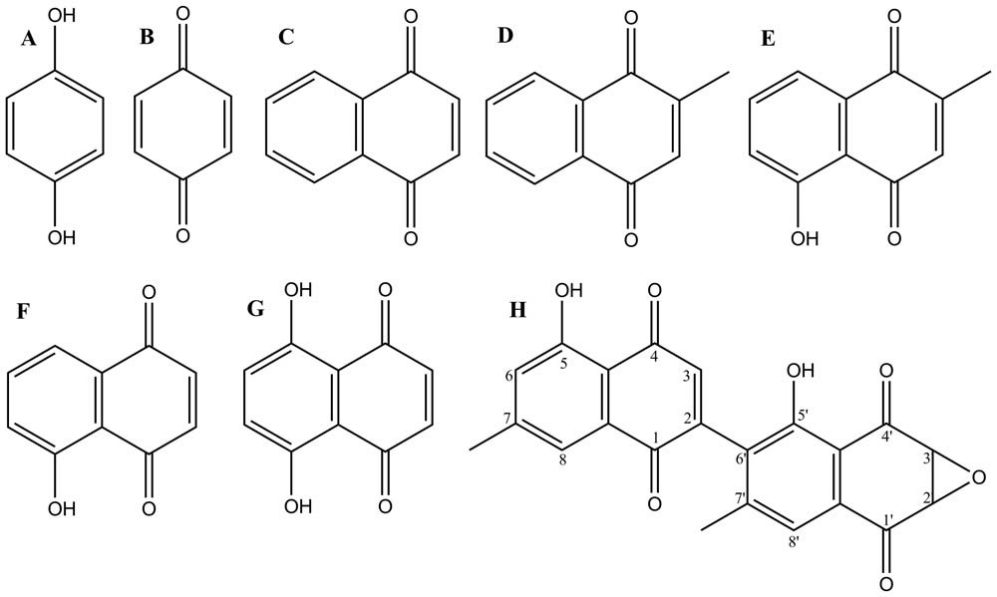
Chemical structures of the tested compounds. (A) *p*-hydroquinone; (B) *p*-benzoquinone; (C) 1,4-naphthoquinone; (D) menadione; (E) plumbagin; (F) juglone; (G) naphthazarin; (H) diosquinone.

In the present work, the ability to induce a decrease of NO in LPS-stimulated RAW 264.7 macrophages of several naphthoquinones (plumbagin, naphthazarin, juglone, menadione, diosquinone and 1,4-naphthoquinone), *p*-hydroquinone and *p*-benzoquinone (Figure 1) was evaluated. This chemical mediator was chosen due to its importance in inflammatory processes and for inflammatory damage, besides being easily quantified. Regarding the anti-inflammatory potential of the involved naphthoquinones, naphthazarin and 1,4-naphthoquinone were already studied for their ability to decrease NO in LPS-stimulated RAW 264.7 macrophages [13]. Nevertheless, naphthoquinones are also known to undergo redox cycling, causing oxidative stress in cells [8]. As far as we know, there are no reports assessing whether the decrease of NO in this model is due to a reduction of its production or to the reaction of NO with superoxide generated during redox cycling of naphthoquinones. If the second hypothesis is true, the generation of peroxynitrite can lead to protein nitration, impairing cellular functioning, and no beneficial effect can be attributed to naphthoquinones. Thus, this work intended to: extend the knowledge on the ability to cause a NO decrease in the LPS – RAW 264.7 macrophages model to other naphthoquinones (juglone, menadione, plumbagin and diosquinone) and related compounds (*p*-hydroquinone and *p*-benzoquinone); establish possible structure-activity relationships; determine if NO decrease induced by naphthoquinones is due to its consumption in peroxynitrite formation. Pro-inflammatory cytokines (TNF-α, IL-1β and IL-6), superoxide radical and protein nitration levels were determined for the most promising compound.

As far as we know, the contribution of oxidative stress induced by naphthoquinones to NO reduction has not been studied yet. Furthermore, diosquinone, an epoxide dimeric naphthoquinone found in several *Diospyros* species [3], [22], was evaluated for its anti-inflammatory potential for the first time herein.

## Materials and Methods

### Materials

DPBS (Dulbecco's Phosphate Buffered Saline), DMEM (Dulbecco's Modified Eagle Medium)+GlutaMAX™-I, heat inactivated foetal bovine serum and penicillin+streptomycin (Pen Strep) were obtained from Gibco, Invitrogen™ (Grand Island, NY, USA). Plumbagin, naphthazarin, juglone, 1,4-naphthoquinone, *p*-hydroquinone, *p*-benzoquinone, menadione, sulphanilamide, potassium nitrite, *β*-nicotinamide adenine dinucleotide reduced disodium salt hydrate (NADH), sodium pyruvate, 3-(4,5-dimethylthiazol-2-yl)-2,5-diphenyltetrazolium bromide (MTT), methanol, triton, tris-HCl, ethylene glycol-bis(2-aminoethylether)-*N,N,N′,N′*-tetraacetic acid (EGTA), brilliant blue G, dimethyl sulfoxide (DMSO), DL-dithiothreitol (DTT), phenylmethanesulfonil fluoride (PMSF), nitrotetrazolium blue chloride (NBT), bovine serum albumin, lipopolysaccharide from *Salmonella enteric* serotype *Typhimurium*, monoclonal anti-3-nitrotyrosine antibody produced in mouse and GBX fixer and developer of Kodak processing chemicals for autoradiography films were purchased from Sigma-Aldrich (St. Louis, MO, USA). *N*-(1)-Naphthylethylenediamine, glycerol and ethylenediaminetetraacetic acid (EDTA) were obtained from Merck (Darmstadt, Germany). Sodium chloride and potassium hydroxide (KOH) were purchased from Vaz Pereira (Lisbon, Portugal) and *ortho*-phosphoric acid 85% was obtained from Panreac (Barcelona, Spain). Goat antibody to mouse IgG (horseradish peroxidase) (0.8 mg/mL), TNF-α, IL-1β and IL-6 mouse ELISA kits were purchased from Abcam (Cambridge, United Kingdom). Hybond – ECL, Hyperfilm™ ECL and the enhanced chemiluminescence (ECL)-Plus reagent Kit were obtained from Amersham™ (GE Healthcare, Piscataway, NJ, USA). Diosquinone was isolated from root barks of *D. chamaethamnus* [3] and its purity was checked by HPLC-DAD.

### Diosquinone HPLC-DAD analysis

Chloroform solution of diosquinone was analysed in a HPLC system (Gilson), using a Spherisorb OSD2 column (Waters, Milford, USA) (250×4.6 mm, i. d., 5 µm). The solvent used was a mixture of acetic acid 5% in water (A) and methanol (B) and the gradient was as follows: 0 min - 75% B, 10 min - 75% B, 25 min - 85% B, 35 min - 100% B and 60 min - 100% B. The HPLC system was equipped with a Gilson diode array detector (DAD). Spectroscopic data of the peak was accumulated in the range of 200–400 nm, and chromatogram was recorded at 255 nm. The data was processed on Unipoint system software (Gilson Medical Electronics, Villiers le Bel, France).

### Cell culture and treatments

The mouse macrophage-like cell line RAW 264.7 was kindly provided by Prof. Maria S. J. Nascimento (Laboratório de Microbiologia, Departamento de Ciências Biológicas, Faculdade de Farmácia, Universidade do Porto). Cells were grown in DMEM+GlutaMAX™ – I supplemented with 10% heat inactivated foetal bovine serum, 100 U/mL penicillin and 100 µg/mL streptomycin, under 5% CO_2_ at 37°C, in humidified air. Cells were plated at 1.5×10^5^ cells/mL in a plate with 48 wells (1 mL/well). The tested compounds were dissolved in DMSO, at 10 mM, and stored, as small aliquots, at −20°C. The compounds were diluted with supplemented DMEM as needed, before cell exposure. When cells reach confluence, the compounds were added, 1 h before exposition to LPS (1 µg/mL). After addition of LPS, the cells were maintained in culture for 18 h. Dexamethasone at a concentration of 50 µM was used as positive control. The effect of all tested compounds in the absence of LPS was also evaluated, in order to observe if they induced changes in NO basal levels. In negative controls, no LPS was added. The final concentration of DMSO was 0.5% (v/v) and all control groups received the same amount of DMSO.

### Lactate dehydrogenase (LDH) leakage assay

The release of the cytosolic enzyme LDH into culture medium was evaluated as follows: an aliquot of culture medium was taken and mixed with a NADH buffered solution and pyruvate solution [23]. LDH activity was measured spectrophotometrically by following the conversion of NADH to NAD^+^, at 340 nm. Results are expressed as a percentage of the respective control (with or without LPS).

### MTT reduction assay

Cellular viability was also assessed by the mitochondria dependent reduction of MTT to formazan, according to Sousa and collaborators [24] with some modifications. After described incubation with tested compounds, culture medium removal and cells washing with DPBS, cells in 48-wells plates were incubated with 1 mL/well of MTT (0.5 mg/mL), during 30 min at 37°C. In the end, supernatant was rejected and formazan was solubilised in DMSO (1 mL). The extent of reduction of MTT to formazan within cells was quantified by measurement of optical density at 550 nm, using a microplate reader (Multiskan ASCENT Thermo®). Results are expressed as a percentage of the respective control (with or without LPS).

### NO determination

In culture, the NO released by the macrophages into the medium is converted to several nitrogen derivatives, from which only nitrite is stable, being easily measured by Griess reagent (1.0% sulphanilamide and 0.1% *N*-(1)-naphthylethylenediamine in 2% phosphoric acid) [13]. After incubation, 100 µL of culture medium supernatant was mixed with the same volume of Griess reagent, during 10 min, at room temperature. The nitrite produced was determined by measuring the optical density at 550 nm, in a microplate reader (Multiskan ASCENT Thermo®). Nitrite was quantified by external standard, using potassium nitrite to generate a standard curve. Results are expressed as a percentage of the control with LPS.

### Measurement of superoxide radical

Superoxide radical was measured by the NBT reduction assay [24]. Each well of a 12-wells plate was seeded with 2 mL of RAW 264.7 macrophages suspension containing 1.5×10^5^ cells/mL. The treatment of cells proceeded as described previously: pre-exposure to tested compounds for 1 h, followed by addition of 1 µg/mL or vehicle and further incubation for 18 h. After incubation, 40 µL of a NBT solution at 1 mg/mL was added to the medium and incubated at 37°C, for 1 h. Then, the incubation medium was removed and cells were lysed with DMSO∶4 mM KOH (1∶1). The absorbance of reduced NBT, formazan, was measured at 630 nm, in a microplate reader (Multiskan ASCENT Thermo®). Results are expressed as a percentage of the control without LPS.

### Protein quantification

Protein quantification was performed by addition of 200 µL of Bradford dye reagent (brilliant blue G, 0.1 mg/mL; ethanol, 5% (v/v); phosphoric acid, 10% (v/v) and water) to 40 µL of samples, pre-diluted 100 times. The photometrical measure was performed at 595 nm. Bovine serum albumin was used to generate a standard curve.

### Slot-immunoblotting analysis of 3-nitrotyrosine

RAW 264.7 macrophages were plated with 4 mL/well at a density of 1.5×10^5^ cells/mL, in a plate with 6 wells. After treatment with dexamethasone (50 µM), diosquinone (1.5 µM) and LPS (1 µg/mL), as described above, the cells were washed and subjected to 200 µL triton lysis buffer (1% triton, 20 mM tris HCl, 150 mM NaCl, 5 mM EGTA, 10 mM EDTA and 10% glycerol) with 1 mM DTT and 0.5 mM PMSF, as proteases inhibitors, during 60 minutes. Samples were centrifuged at 12 000 rpm, for 10 minutes, at 4°C, and respective supernatants were collected. Samples solutions were diluted to contain the same protein concentration (0.5 mg/mL) and 100 µL of these solutions were applied in a nitrocellulose membrane, pre-washed with 10% of methanol and pre-hydrated during 5 min. Negative pressure was applied after application of all samples (in duplicate). The membrane was then disassembled from the apparatus and incubated in a TBS-T blocking solution with 5% milk for 5 h, at room temperature. A mouse polyclonal anti-3-nitrotyrosine antibody was used at a dilution of 1∶1000 in TBS-T blocking solution with 5% milk. The membrane and the antibody were incubated overnight, at 4°C, and further incubated at room temperature for 2 h. After washing the membrane with TBS-T, during five minutes, three times, a goat anti-mouse secondary antibody conjugated with horseradish peroxidase (1∶2000) was applied to the membrane for 2 h, at room temperature. All incubations were made with mixing. Finally, the membrane was washed three times with TBS-T buffer and one time with TBS. Blots were revealed using the ECL system kit and 3-nitrotyrosine were visualized on high performance chemiluminescence film, after 15 min of exposition of film to membrane. Three assays were performed. This method is in accordance to Moreira-Gonçalves and collaborators with some modifications [25]. Three-dimensional surface plot analyses were generated with the “Surface Plot” function of *Image J* 1.44 (http://rsbweb.nih.gov/ij/download.html).

### Enzyme linked immunosorbent assay (ELISA)

RAW 264.7 macrophages were plated with 4 mL/well at a density of 1.5×10^5^ cells/mL, in a 6 wells plate. Supernatants from dexamethasone (50 µM), diosquinone (1.5 µM) exposed macrophages, either without or with LPS (1 µg/mL) stimulation, were collected at 18 h and centrifuged at 4000 rpm for 3 min, to remove any cells. Cell free culture media were stored at −70°C until use. TNF-α, IL-1β and IL-6 secreted cytokines were measured in duplicate, using purified biotinylated antibodies in ELISA sets according to the protocol provided by the supplier (Abcam, Cambridge, United Kingdom). The ELISA plates were read using a microplate reader (Multiskan ASCENT Thermo®).

### Statistical analysis

OneWay ANOVA and Dunnett test, as post-hoc test, were used to determine the statistical significance in comparison to control. Data are expressed as the mean ± standard error of the mean (SEM) of at least four independent experiments, performed in duplicate or triplicate, as described above. *P* values of 0.05 or less were considered statistically significant. Statistical analysis was made using: PAWS Statistic 18 Software (Chicago, IL, USA) and Graphpad Prism 5 Software (San Diego, CA, USA).

## Results

### Cellular viability

**Figure 2:**
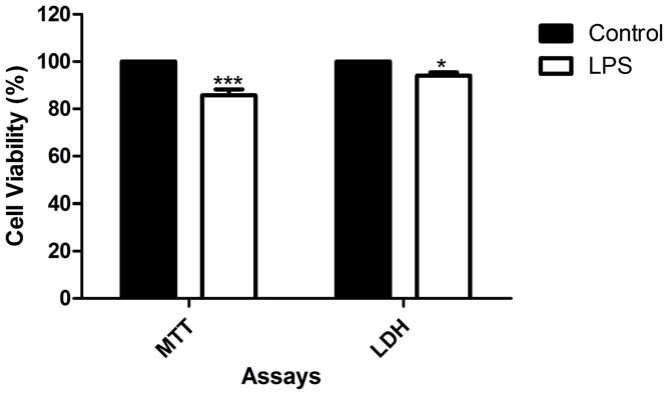
Influence of LPS in cell viability. RAW 264.7 macrophages were exposed to 1 µg/mL of LPS, during 18 h and cell viability were assessed by LDH and MTT assays. Results are expressed in percentage of control (mean ± SEM of five independent experiments, performed in duplicate). *P<0.05, ***P<0.001.

**Figure 3:**
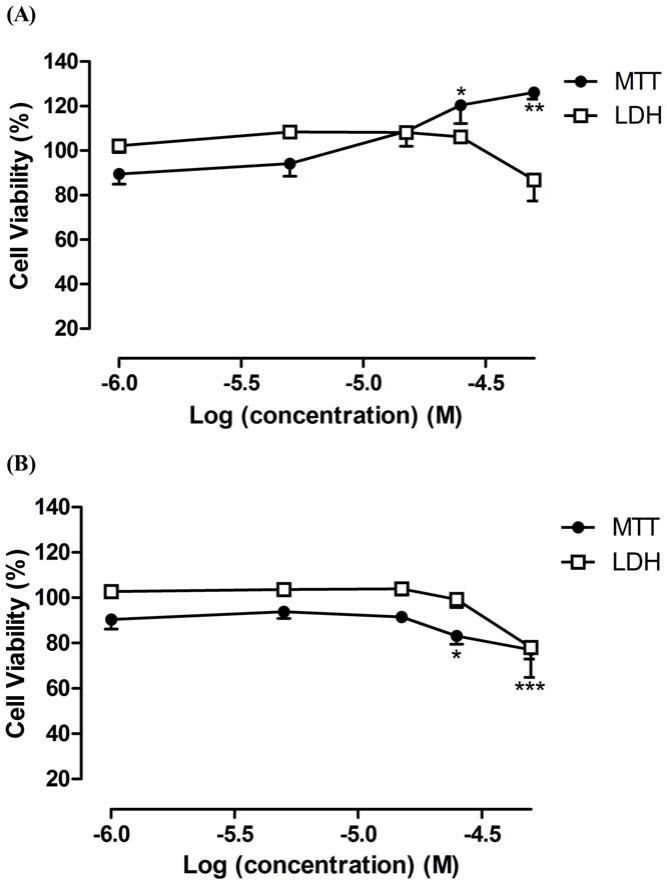
Influence of dexamethasone with and without LPS in cell viability. RAW 264.7 macrophages were pre-exposed for 1 h to dexamethasone followed by 18 h co-exposition with 1 µg/mL of LPS (A) or vehicle (B). The cell viability was assessed by MTT and LDH assays. All results are percentage of control (mean ± SEM of five independent experiments, performed in duplicate). *P<0.05, **P<0.01, ***P<0.001.

The exposure to LPS (1 µg/mL) for 18 h induced a decrease in cellular viability (14.22±2.50% relative to control, in MTT assay), with statistical importance (*p*<0.001) (Figure 2). Dexamethasone (1–50 µM) was used as positive control. At 50 µM, dexamethasone alone caused 22.98±4.11% (*p*<0.001) of cell death, as evaluated by MTT assay. However, for the same concentration of dexamethasone and in the presence of LPS, cell viability of RAW 264.7 macrophages was higher than 100% relative to control (*p*<0.01). These differences in cell viability were not verified in the LDH assay (Figure 3). Therefore, as MTT assay allowed detecting more alterations on cells' survival than the measure of LDH leakage (Figures 2 and 3), the results presented herein for cell viability after treatments with naphthoquinones were obtained by the first assay.

**Figure 4:**
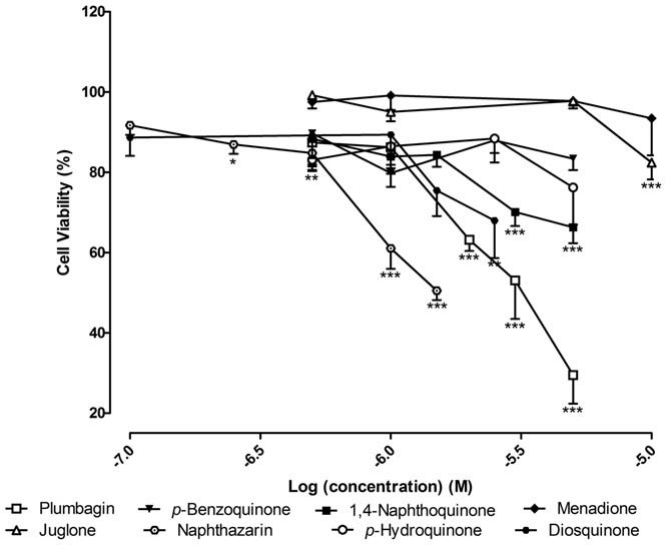
Influence of tested compounds in cell viability. Viability of LPS stimulated RAW 264.7 macrophages, after 19 h of exposure to the tested compounds, by MTT assay. All results are expressed in percentage of control with LPS (mean ± SEM of four independent experiments, performed in duplicate). *P<0.05, **P<0.01, ***P<0.001.

**Figure 5:**
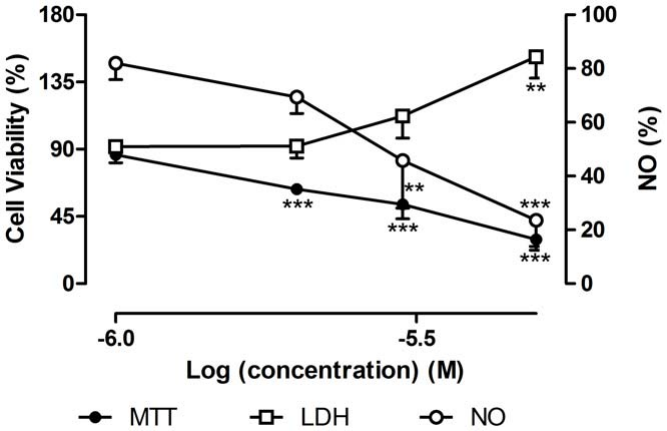
Influence of plumbagin in cell viability and in NO production. After pre-exposure with plumbagin and stimulation with LPS, RAW 264.7 macrophages viability was assessed by LDH and MTT assays and NO production was quantified. Results are expressed as percentage of control (mean ± SEM of four independent experiments, performed in duplicate).**P<0.01, ***P<0.001.

Naphthazarin was the most toxic compound (LC_25_ = 0.73±0.07 µM), followed by plumbagin (LC_25_ = 1.26±0.28 µM) (Figure 4). For plumbagin, at 5 µM, there was 70.50±7.12% (*p*<0.001) of cell death, by MTT assay, while in the LDH assay cell viability was higher than 100% (*p*<0.01) (Figure 5). In order to explore this result, a study of cell viability over time, with 5 µM of plumbagin was performed (data not shown). In the MTT assay, it was verified that cell death began to occur 2 h after treatment, rapidly increasing until 5 h and reaching 70% at the end of the treatment (19 h). Cell death measured by LDH assay showed the same profile during the first 8 h. After this period, the LDH quantified in the culture medium began to decrease. Cell death overtime was confirmed by microscopic evaluation.

1,4-Naphthoquinone (*p*<0.001) and diosquinone (*p*<0.01) showed some toxicity at concentrations of 5 µM and 2.5 µM, respectively (about 35% of cell death relative to control). Juglone caused nearly 20% of cell death at 10 µM (*p*<0.001). Menadione (0.5–10 µM), *p*-hydroquinone and *p*-benzoquinone (0.5–5 µM) revealed no cytotoxicity under the concentration range used (Figure 4).

### NO production

None of the tested compounds induced changes in NO basal levels, when incubated without LPS (data not shown).

**Figure 6:**
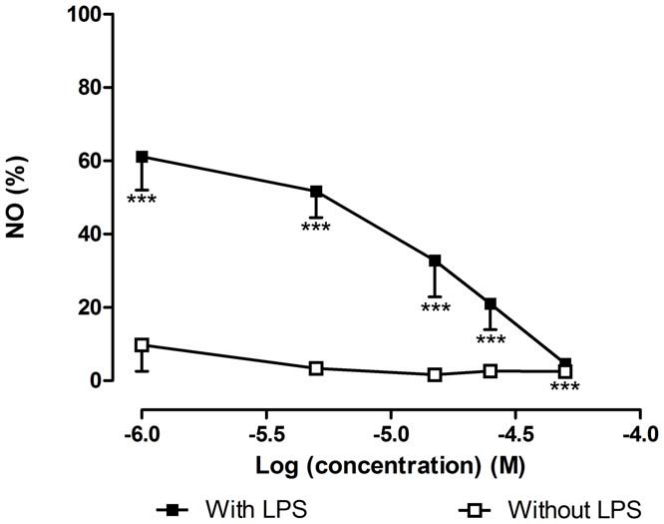
Dexamethasone effects on NO production. Quantification of NO produced by RAW 264.7 macrophages exposed to dexamethasone, in the presence and in the absence of 1 µg/mL of LPS (18 h). All results are expressed in percentage of control with LPS (mean ± SEM of four independent experiments, performed in duplicate). ***P<0.001.

Dexamethasone caused a decreased of NO for all tested concentrations, having an IC_25_ of 1.72±0.54 µM. With 50 µM of dexamethasone, the amount of NO was similar to basal levels (Figure 6).

**Figure 7:**
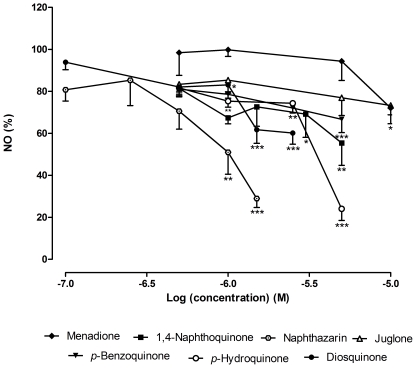
Influence of tested compounds in NO production. Quantification of NO produced by LPS stimulated RAW 264.7 macrophages after pre-exposure to tested compounds. Results are expressed in percentage of control (0.5% DMSO+1 µg/mL LPS) (mean ± SEM of four independent experiments, performed in duplicate). *P<0.05, **P<0.01, ***P<0.001.

Diosquinone decreased NO to 38.25±6.50% (*p*<0.001) relative to control at 1.5 µM, with an IC_25_ of 1.09±0.24 µM (Figure 7). The IC_25_ found for 1,4-naphthoquinone was 0.72±0.09 µM. However, at 1.5 µM, NO reduction by 1,4-naphthoquinone was 27.36±0.28% (*p*>0.05). Menadione did not induce NO reduction with statistical significance. Juglone, naphthazarin and plumbagin did not cause a decrease of NO at non cytotoxic concentrations (Figures 4, 5 and 7).

*p*-Benzoquinone and *p*-hydroquinone exhibited an IC_25_ of 1.82±0.57 µM and 1.14±0.28 µM, respectively, being the last more active than *p*-benzoquinone: *p*-hydroquinone caused a reduction of 50% of NO, at 3.74±0.18 µM (Figure 7).

As diosquinone caused higher NO reduction than the other tested naphthoquinones to lower concentrations, it was chosen to study the effect on superoxide and pro-inflammatory cytokines production and protein nitration.

### Superoxide generation

**Figure 8:**
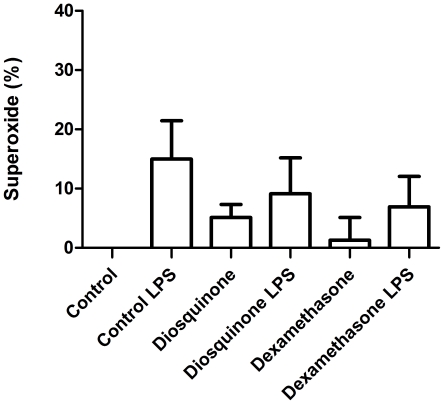
Superoxide radical quantification. Superoxide radical produced by RAW 264.7 macrophages, after pre-treatment (1 h) with diosquinone (1.5 µM) and dexamethasone (50 µM), in the presence and in the absence of 1 µg/mL of LPS (18 h). Results are expressed in percentage of control (mean ± SEM of four independent experiments, performed in duplicate).

The different treatments (without tested compounds, with 1.5 µM of diosquinone and 50 µM of dexamethasone, in the presence and in the absence of LPS) did not reveal significant differences (Figure 8). However, LPS seemed to increase superoxide generation, especially in control cells.

### Protein nitration

**Figure 9:**
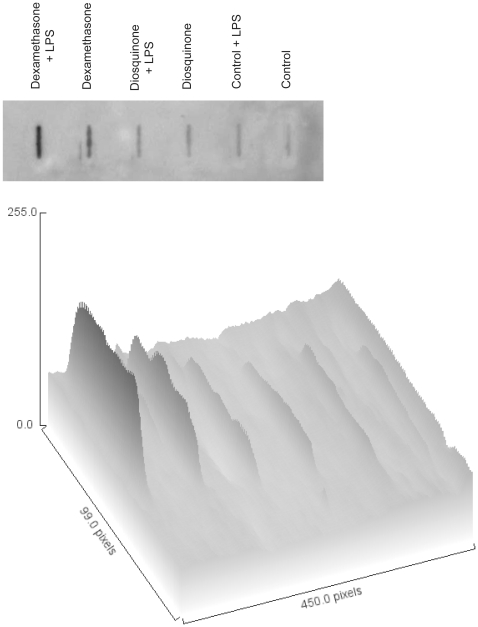
Semi-quantitative analysis of 3-nitrotyrosine. Image of slot-blot film, where 3-nitrotyrosine was detected by immunoblotting and respective surface plot analysis (*Y* axis represents intensity of bands). This assay was performed after RAW 264.7 macrophages pre-treatment (1 h) with diosquinone (1.5 µM) and dexamethasone (50 µM) in presence and in absence of 1 µg/mL of LPS (18 h). Control cells were exposed to vehicle (0.5% DMSO).

Diosquinone (1.5 µM) alone did not increase protein nitration, relative to control. On the other hand, dexamethasone (50 µM) by itself led to protein nitration, which was potentiated by LPS (Figure 9).

### Pro-inflammatory cytokines

Diosquinone (1.5 µM) was able to reduce TNF-α by 51.38±9.72% and IL-6 by 39.42±19.79% relative to LPS exposed cells only. With respect to IL-1β, macrophages stimulation with LPS did not cause a measurable increase of this cytokine under the assay conditions. So, no reduction in this interleukin could be observed in cells exposed to diosquinone and LPS.

## Discussion

Several compounds, the majority of them being naphthoquinones (Figure 1), were screened for their ability to alter NO production by LPS stimulated RAW 264.7 macrophages. In a second part of this work, assays were made to confirm the beneficial effect of diosquinone, the most promising one. Diosquinone, previously isolated from *D. chamaethamnus* [3], was pure, as ascertained by HPLC-DAD analysis (data not shown).

Macrophages, which express high levels of inducible NO synthase, play a central role in host's defence against bacterial infection, being the major cellular targets for LPS action [26].

Among inflammatory modulators produced in response to LPS, NO is an important cytotoxic mediator [27]. The first precaution when studying the direct interference of a given compound on NO production is to guarantee that it does not cause cell death, decreasing the number of NO producing cells. Thus, the effect on cell viability should be assessed.

The results obtained after incubation with dexamethasone in MTT assay depended on the presence of LPS (Figure 3): when cells were treated with both LPS and dexamethasone the cellular viability was higher than 100% (*p*<0.01), while the treatment only with dexamethasone gave the opposite result. This could be explained by the mechanism of action of dexamethasone, which inhibits NF-κB transcription factor [28]. NF-κB is central to a series of cellular processes, like inflammation, cell proliferation and apoptosis [29]. As constitutive expression of NF-κB is frequently found in tumour cells [30], the NF-κB inhibitory action of dexamethasone can be responsible for the decrease in RAW 264.7 macrophages viability. As LPS activates NF-κB [20] and dexamethasone has an opposite activity, the dexamethasone effect in cell survival is counterbalanced by the presence of LPS.

In this work, LDH and MTT assays revealed different effects on cellular viability. The decrease in cell survival, in the presence of 50 µM of dexamethasone, was not detected by LDH assay (Figure 3). Analogous observations had also been reported by other authors [23], [31]. LDH assesses cell death due to cell membrane damage which leads to the release of LDH into culture medium [23]. MTT assay provides information on mitochondrial function, as MTT reduction mainly occurs in this organelle through the action of succinate dehydrogenase [23]. The MTT assay is also a preferential method when cell death occurs long before cellular viability determination, as we observed for plumbagin treatment at 5 µM. With this naphthoquinone, cell viability after 19 h exposition assessed by the MTT assay was too low, while it reached a mean of 151.90±41.39% (*p*<0.01) in the LDH assay (Figure 5). Therefore, the influence of exposition time on cell viability was studied. It was observed that cell death occurred mainly in the beginning of the exposition period (data not shown). Thus the majority of the LDH leaked to the medium was degraded at 19 h. In this study, MTT assay appeared to be more sensitive in detecting toxicity compared to the LDH leakage assay. For these reasons, the MTT assay was chosen to assess the effect on cell viability.

Another important factor was the choice of the LPS concentration to be used in the assays. The LPS concentration must be such that not causes cell death, but allows a measurable NO amount. In this work, 1 µg/mL of LPS was used, which is a concentration currently applied by other authors [13], [32]. However, the exposition of RAW 264.7 macrophages to 1 µg/mL of LPS for 18 hours lead to 14.22±2.50% (*p*<0.001) of cell death relative to control (Figure 2). This result can be explained by excessive reactive oxygen species production (about 15% increase in superoxide relative to control), as it can be seen in Figure 8. As so, the results of cell viability in the presence of LPS for the tested compounds are expressed in percentage of control with LPS. In addition, LPS (1 µg/mL) induced NO production in amounts easy to quantify (19.21±0.274 µM) and we considered that NO produced by LPS exposed RAW 264.7 cells was 100%.

Concerning the cytotoxicity of tested compounds, naphthazarin and plumbagin were the most toxic ones (Figure 4). In general, tumour cells, as RAW 264.7 macrophages, are sensitive to the deleterious effects of naphthoquinones in low µM range. In studies involving other tumour and non-tumour cells [10], [30], the authors used plumbagin concentrations similar to those used in our work, but no significant cell death was noticed. This difference may be explained by distinct sensitivity of the cells to naphthoquinones.

The cytotoxicity of diosquinone was lower compared to most of the other tested hydroxy-naphthoquinones (Figure 4). Quinones cytotoxicity is related with their pro-oxidant properties and interaction with nucleophilic biomolecules [33], [34]. It is known that nucleophilic attack to thiol groups involves positions 2 and 3 of naphthoquinones [35]. As diosquinone has an epoxy group at positions 2 and 3 of one monomer and the C2 of the other monomer is involved in the linkage between the two monomers, probably diosquinone causes less oxidative stress and glutathione depletion than the other naphthoquinones. For the other compounds, the presence of a hydroxyl group in the benzene ring seems to be important for the exhibited toxicity, as the introduction of electron-donating hydroxyl groups increases the pro-oxidant potential of naphthoquinones [33], [34]. This may explain the higher toxicity of naphthazarin and plumbagin and why juglone is more toxic than menadione. However, juglone is less toxic than the corresponding non-hydroxylated 1,4-naphtoquinone, which is in accordance with a previous work by Klaus and colleagues [33]. In addition, hydroxyl groups at positions 5 and 8 of naphthazarin confer a high redox potential, explaining its higher toxicity relative to other naphthoquinones [8].

In what concerns to NO decrease (Figure 7), it seemed to follow the effect on cell viability (Figure 4). However, the decrease of NO was, in general, more pronounced. Furthermore, we only concluded about NO reduction if no cell death with statistical meaning occurred. Diosquinone, the only tested dimer, was the most interesting compound. Diosquinone caused 38.25±6.50% (*p*<0.001) of NO reduction relative to control at 1.5 µM (Figure 7). Although *p*-hydroquinone caused 75.92±5.53% (*p*<0.001) of NO reduction at 5 µM, NO reduction for 3.20±0.16 µM corresponded to 40%. Thus, diosquinone was more active at lower concentrations than *p*-hydroquinone. Diosquinone had an IC_25_ of 1.09±0.24 µM, lower than that of dexamethasone (1.72±0.54 µM). However, dexamethasone caused a decrease in NO for all used concentrations, reaching basal levels at 50 µM (Figure 6). The greater activity of diosquinone in relation to the other naphthoquinones could be explained by its higher lipophilicity. Lipophilicity favours both the entrance of the compound in the cell and the establishment of hydrophobic bonds with a potential active site [36]. The importance of lipophilicity may also be observed when comparing the activities of 1,4-naphthoquinone and *p*-benzoquinone: 1,4-naphthoquinone was more cytotoxic and lead to a higher NO decrease than *p*-benzoquinone (Figures 4 and 7).

Although naphthoquinones may suppress NF-κB activation and, consequently, inhibit iNOS induction [13], [30], the observed NO reduction can also result from direct reaction of NO with superoxide radical, which is generated during quinone oxidation [37]. The product of this reaction is peroxynitrite, which may react with thiol groups or nitrate and hydroxylate phenolic amino acids, most importantly tyrosine residues, forming 3-nitrotyrosine. The increase of 3-nitrotyrosine has toxicological consequences, because it alters phosphorylation of tyrosine [38]. Therefore, to clarify the effect of diosquinone we proceeded to the determination of superoxide radical and 3-nitrotyrosine.

It was observed that diosquinone (at 1.5 µM) did not induce more nitration than control (Figure 9), indicating that the decrease of NO is beneficial to cells, precluding LPS action and reducing inflammation. Some authors defend that nitration of tyrosine may not occur, having nitration of dityrosine [39], [40]. However, that seemed to be not the case, as the used method allowed to detect high levels of 3-nitrotyrosine with dexamethasone and LPS treatment. With respect to superoxide radical, no significant differences were noticed, although cells treated with LPS tended to exhibit higher levels of this reactive species (Figure 8).

The potential of diosquinone to reduce LPS induced inflammation was confirmed by its ability to reduce the pro-inflammatory cytokines TNF-α and IL-6, which have a central role in inflammation. The decrease in TNF-α was the most expressive and since this cytokine has a fundamental role in the activation of macrophages themselves, being the first mediator in the inflammatory cascade [21], diosquinone may have interest to be explored as candidate to a new anti-inflammatory drug.

In conclusion, among the several compounds screened for anti-inflammatory activity, diosquinone, the only dimeric naphthoquinone, was the most promising naphthoquinone, causing a reduction of NO, without cytotoxicity. The NO decrease induced by this compound was probably due to its interference with the mechanism of action of LPS and not to NO consumption in the reaction with superoxide. This hypothesis was confirmed by the reduction of the inflammatory mediators TNF-α and IL-6 in macrophages stimulated with LPS. Thus, according to these results, diosquinone brings beneficial effects to cells by inhibiting the inflammatory response. This study contributes to the knowledge of naphthoquinones properties, mainly anti-inflammatory activity, and, as far as we are aware, constitutes the first work concerning diosquinone anti-inflammatory potential. Furthermore, it is the first study assessing whether the oxidative stress induced by naphthoquinones contributes to NO reduction, in this model.

## References

[1] Babula P, Adam V, Havel L, Kizek R (2009). Noteworthy secondary metabolites naphthoquinones – their occurrence, pharmacological properties and analysis.. Curr Pharm Anal.

[2] Mallavadhani UV, Panda AK, Rao YR (1998). Pharmacology and chemotaxonomy of *Diospyros*.. Phytochemistry.

[3] Costa MA, Alves AC, Seabra RM, Andrade PB (1998). Naphthoquinones of *Diospyros chamaethamnus*.. Planta Med.

[4] Adeniyi BA, Fong HHS, Pezzuto JM, Luyengi L, Odelola HA (2000). Antibacterial activity of diospyrin, isodiospyrin and bisisodiospyrin from the root of *Diospyros piscatorial* (Gurke) (Ebenaceae).. Phytother Res.

[5] Theerachayanan T, Sirithunyalug B, Piyamongkol S (2007). Antimalarial and antimycobacterial activities of dimeric naphthoquinones from *Diospyros glandulosa* and *Diospyros rhodocalyx*.. CMU J Nat Sci.

[6] Hernández-Pérez M, Rabanal RM, de la Torre MC, Rodríguez B (1995). Analgesic, anti-inflammatory, antipyretic and haematological effects of aethiopinone, an *o*-naphthoquinone diterpenoid from *Salvia aethiopis* roots and two hemisynthetic derivatives.. Planta Med.

[7] Chakrabarty S, Roy M, Hazra B, Bhattacharya RK (2002). Induction of apoptosis in human cancer cell lines by diospyrin, a plant-derived bisnaphthoquinonoid, and its synthetic derivatives.. Cancer Lett.

[8] O'Brien PJ (1991). Molecular mechanisms of quinone cytotoxicity.. Chem Biol Interact.

[9] Kuke C, Williamson EM, Roberts MF, Watt R, Hazra B (1998). Antiinflammatory activity of binaphthoquinones from *Diospyros* species.. Phytother Res.

[10] Checker R, Sharma D, Sandur SK, Khanem S, Poduval TB (2009). Anti-inflammatory effects of plumbagin are mediated by inhibition of NF-kappaB activation in lymphocytes.. Int Immunopharmacol.

[11] Subbaramaiah K, Bulic P, Li Y, Dannenberg AJ, Parco DS (2001). Development and use of a gene promoter-based screen to identify novel inhibitors of cyclooxygenase-2 transcription.. J Biomol Screen.

[12] Wang JP, Raung SL, Chang LC, Kuo SC (1995). Inhibition of hind-paw edema and cutaneous vascular plasma extravasation in mice by acetylshikonin.. Eur J Pharmacol.

[13] Cheng YW, Chang CY, Lin KL, Hu CM, Lin CH (2008). Shikonin derivatives inhibited LPS-induced NOS in RAW 264.7 cells via downregulation of MAPK/NF-κB signaling.. J Ethnopharmacol.

[14] Han A-R, Min H-Y, Nam J-W, Lee N-Y, Wiryawan A (2008). Identification of a new naphthalene and its derivatives from the bulb of *Eleutherine americana* with inhibitory activity on lipopolysaccharide-induced nitric oxide production.. Chem Pharm Bull.

[15] Moncada S, Plamer RMJ, Higgs EA (1991). Nitric oxide: physiology, pathophysiology and pharmacology.. Pharmacol Rev.

[16] Swett MJ, Hume DA (1996). Endotoxin signal transduction in macrophages.. J Leukoc Biol.

[17] Andreakos ET, Foxwell BM, Brennan FM, Maini RN, Feldmann M (2002). Cytokines and anti-cytokine biological in autoimmunity: present and future.. Cytokine Growth Factor Rev.

[18] El-Omar EM, Carrington M, Chow W-H, Kenneth EL, McColl KEL (2000). Interleukin-1 polymorphisms associated with increased risk of gastric cancer.. Nature.

[19] Stein B, Sutherland MSK (1998). IL-6 as a drug discovery target.. Drug Discov Today.

[20] Maeda S, Omata M (2008). Inflammation and cancer: Role of nuclear factor-kappaB activation.. Cancer Sci.

[21] Laskin DL, Pendino KJ (1995). Macrophages and inflammatory mediators in tissue injury.. Ann Rev Pharmacol Toxicol.

[22] Alves AC, Costa MA, Paul MI (1983). Naphthoquinones of *Diospyros batocana*.. Planta Med.

[23] Lobner D (2000). Comparison of the LDH and MTT assays for quantifying cell death: validity for neuronal apoptosis?. J Neurosci Methods.

[24] Sousa C, Pontes H, Carmo H, Dinis-Oliveira RJ, Valentão P (2009). Water extracts of *Brassica oleracea* var. *costata* potenciate paraquat toxicity to rat hepatocytes *in vitro*.. Toxicol in Vitro.

[25] Moreira-Gonçalves D, Henriques-Coelho T, Fonseca H, Ferreira RM, Amado F (2011). Moderate exercise training provides left ventricular tolerance to acute pressure overload.. Am J Physiol Heart Circ Physiol.

[26] Adams DO, Hamiltonm TA (1984). The cell biology of macrophage activation.. Ann Rev Immunol.

[27] Nathan CF, Hibbs JB (1991). Role of nitric oxide synthesis in macrophage antimicrobial activity.. Curr Opinion Immunol.

[28] De Vera ME, Taylor BS, Wang Q, Shapiro RA, Billiar TR (1997). Dexamethasone suppresses iNOS gene expression by upregulating IkB-α and inhibiting NF-κB.. Am J Physiol Gastrointest Liver Physiol.

[29] Sun Z, Andersson R (2002). NF-κB activation and inhibition: a review.. Shock.

[30] Sandur SK, Ichikawa H, Sethi G, Ahn KS, Aggarwal BB (2006). Plumbagin (5-hydroxy-2-methyl-1,4-naphthoquinone) suppresses NF-κB activation and NF-κB-regulated gene products through modulation of p65 and IκBα kinase activation, leading to potentiation of apoptosis induced by cytokine and chemotherapeutic agents.. J Biol Chem.

[31] Fotakis G, Timbrell JA (2005). *In vitro* cytotoxicity assays: Comparison of LDH, neutral red, MTT and protein assay in hepatoma cells lines following exposure to cadmium chloride.. Toxicol Lett.

[32] Chia JKS, Pollack M, Guelde G, Koles NL, Miller M (1989). Lipopolysaccharide (LPS) - Reactive monoclonal antibodies fail to inhibit LPS-induced tumor necrosis factor secretion by mouse-derived macrophages.. J Infect Dis.

[33] Klaus V, Hartmann T, Gambini J, Graf P, Stahl W (2010). 1,4-Naphthoquinones as inducers of oxidative damage and stress signalling in HaCaT human keratinocytes.. Arch Biochem Biophys.

[34] Murakami K, Haneda M, Iwata S, Yoshino M (2010). Effect of hydroxyl substituent on the prooxidant action of naphthoquinone compounds.. Toxicol in Vitro.

[35] Inbaraj JJ, Chignell CF (2004). Cytotoxic action of juglone and plumbagin: A mechanistic study using HaCaT keratinocytes.. Chem Res Toxicol.

[36] Liu X, Chuman H (2005). Determination of solute lipophilicity by reversed-phase high-performance liquid chromatography (RP-HPLC).. J Med Invest.

[37] Gryglewski RJ, Palmer RMJ, Moncada S (1986). Superoxide anion is involved in the breakdown of endothelium-derived vascular relaxing factor.. Nature.

[38] Mallozzi C, Di Stasi AMM, Minetti M (1997). Peroxynitrite modulates tyrosine-dependent signal transduction pathway of human erythrocyte band 3.. FASEB J.

[39] Pfeiffer S, Mayer B (1998). Lack of tyrosine nitration by peroxynitrite generated at physiological pH.. J Biol Chem.

[40] Pfeiffer S, Schmidt K, Mayer B (2000). Dityrosine formation outcompetes tyrosine nitration at low steady-state concentrations of peroxynitrite.. J Biol Chem.

